# Ebola and Marburg Viruses: A View of Infection Using Electron Microscopy

**DOI:** 10.3201/eid1008.040350

**Published:** 2004-08

**Authors:** Pierre Rollin

**Affiliations:** * Centers for Disease Control and Prevention, Atlanta, Georgia, USA

**Keywords:** Ebola, Marburg Virus, electron microscopy

More than 25 years have passed since the discovery of a filovirus, Marburg virus, which caused an epidemic of hemorrhagic fever among laboratory workers in Marburg, Germany, in 1967. The persons affected had contact with the blood or tissues of monkeys or with other infected persons. Marburg virus has reappeared only three times since its discovery, with the largest and most recent outbreak occurring in 1999 in Durba, Democratic Republic of the Congo. Ebola virus, another filovirus, was first described in 1976 during two hemorrhagic fever epidemics in Zaire and Sudan. Since then, Ebola virus has caused large hospital outbreaks of hemorrhagic fever in Kikwit, Zaire, in 1995, and Gulu, Uganda, in 2000. Ebola virus has also been implicated in small chains of transmission among persons with direct contact with intermediary hosts, mostly nonhuman primates in the central African countries of Gabon and Republic of the Congo.

The reservoirs for both viruses are still unknown, and the rarity of outbreaks and the remote location of human outbreaks make it difficult, if not impossible, to study the pathogenesis of the human disease. Thus, animal models have been the best, and often only, approach available for studying the progression of disease caused by Marburg and Ebola viruses. Dr. Ryabchikova, the principal author of this book, and her laboratory group have studied the pathogenesis of filoviruses for several decades by using animal models and electron microscopy, a unique approach that has made her one of the few filovirus experts in the world. This book is a compilation not only of her work but of all the information available on Marburg and Ebola viruses.

The first three chapters of the book provide a general review of filovirus history, laboratory methods (with an emphasis on electron microscopy), viral structure, morphology, and replication. Chapters 4 and 5 provide more specific details on infection of the target cells (macrophages and reticuloendothelial system) in different organs and during the course of filoviral infection. In chapter 6, the authors deal with the "hemorrhagic" side of Ebola and Marburg virus infection. Not all patients infected with these viruses bleed, and when bleeding disorders do occur, no correlated infection of endothelial and hematopoietic cells occurs. Dr. Ryabchikova has found that changes in the microcirculation system, such as the appearance of hemorrhages, clotting, and fibrin deposits, vary by virus and by animal species. Chapter 7, which describes pathologic changes in the organs during the course of filoviral infection, could have been combined with chapter 5. Likewise, the last chapter, which covers immunopathology, appears more like a discussion of the previous chapters.

Much of the data have already been published in the Russian or Western literature. However, this book provides one source for all information available on Marburg and Ebola viruses and has a great advantage over other sources. The number and the quality of the illustrations are impressive, and a comprehensive index is provided. The book will prove useful to clinicians and researchers interested in understanding the pathogenesis of hemorrhagic fevers, and it will provide researchers working with other viruses a lesson in the benefits of using electron microscopy technology.

